# Awake Fiberoptic Intubation for Severe Tracheal Compression Caused by a Multinodular Goiter: A Case Report

**DOI:** 10.7759/cureus.101150

**Published:** 2026-01-09

**Authors:** Rita Leite Aguiar, Patrícia Santos, Amélia Ferreira

**Affiliations:** 1 Anesthesiology, Centro Hospitalar e Universitário de São João, Porto, PRT

**Keywords:** airway management, difficult airway management, severe tracheal compression, substernal goiter, tracheal compression, tracheal stenosis

## Abstract

Massive multinodular goiters may cause critical tracheal compression and distortion of anterior neck anatomy, rendering both conventional airway management and front-of-neck access (FONA) unsafe. Awake fiberoptic intubation (AFOI) is often recommended, but failure may still occur, requiring reassessment and alternative rescue strategies.

We report a 53-year-old male with a long-standing multinodular goiter causing severe tracheal narrowing (minimum diameter, 6.2 mm) and marked deviation. An initial AFOI attempt failed due to inadequate tolerance and airway collapse. Following multidisciplinary reassessment and optimization of topical anesthesia and airway strategy, a second AFOI was successfully performed. General anesthesia and thyroidectomy proceeded uneventfully. Extubation was deferred due to concern for postoperative secondary tracheomalacia. Following airway assessment and staged weaning, the patient was extubated over an airway exchange catheter and recovered without complications.

This case highlights the importance of structured decision-making in anticipated difficult airway scenarios where FONA is not feasible. It emphasizes that failed AFOI should prompt reassessment rather than abandonment of the awake approach and that extubation represents a second high-risk airway intervention requiring planning. The role of extracorporeal membrane oxygenation (ECMO) as a rescue strategy in extreme cases is discussed.

In patients with severe goiter-related tracheal compression, successful airway management relies on multidisciplinary planning, optimization of awake intubation technique, and a staged extubation strategy supported by airway exchange devices.

## Introduction

Tracheal deviation and luminal narrowing caused by a markedly enlarged thyroid gland create a complex and high-risk airway scenario that demands careful preprocedural planning. Conventional difficult airway algorithms offer limited guidance when front-of-neck access (FONA), including cricothyrotomy or tracheostomy, is rendered unsafe or impossible by distorted anatomy. Awake fiberoptic intubation (AFOI) is generally regarded as the gold standard for an anticipated difficult airway, as it preserves spontaneous ventilation and intrinsic airway tone until the airway is definitively established [1]. However, even AFOI may fail when tracheal compression is extreme or when stenosis involves long or distal segments of the airway.

In patients with large multinodular or substernal goiters, airway compromise may be further exacerbated by dynamic airway collapse, reduced tracheal compliance, and obliteration of normal cervical landmarks, significantly increasing the risk of airway obstruction following induction of general anesthesia. Recent airway management guidelines emphasize that when both mask ventilation and emergency FONA are predicted to be difficult or impossible, awake techniques that maintain spontaneous ventilation should be prioritized [1,2].

Computed tomography (CT) plays a central role in preoperative risk stratification by allowing objective assessment of tracheal diameter, length of compression, and degree of deviation, features that have been associated with increased airway risk [2,3].

In this case report, we describe a patient with a massive substernal goiter causing extreme narrowing of the upper trachea who underwent successful awake nasotracheal fiberoptic intubation after an initial failed attempt. We additionally discuss the potential role of venovenous extracorporeal membrane oxygenation (ECMO) as a rescue modality when all conventional airway strategies are predicted to fail.

## Case presentation

A 53-year-old male patient (ASA physical status II), with a history of obesity (BMI of 31 kg/m²), medically optimized hypertension and dyslipidemia, active smoking, and obstructive sleep apnea under continuous positive airway pressure (CPAP), was scheduled for elective total thyroidectomy for a long-standing multinodular goiter. He reported a painless, progressively enlarging cervical mass over a two-year period. During the preceding six months, he developed hoarseness and an intermittent dry cough. He denied dysphagia or orthopnea and was able to tolerate the supine position.

Airway examination revealed a large anterior neck mass with significant tracheal deviation. Cervical mobility was mildly restricted. Mallampati classification was II, thyromental distance exceeded 6.5 cm, and the upper lip bite test was grade 1. No venous engorgement or ophthalmopathy was present. Anterior neck landmarks were poorly defined, raising concern regarding the feasibility of emergency FONA.

**Figure 1:**
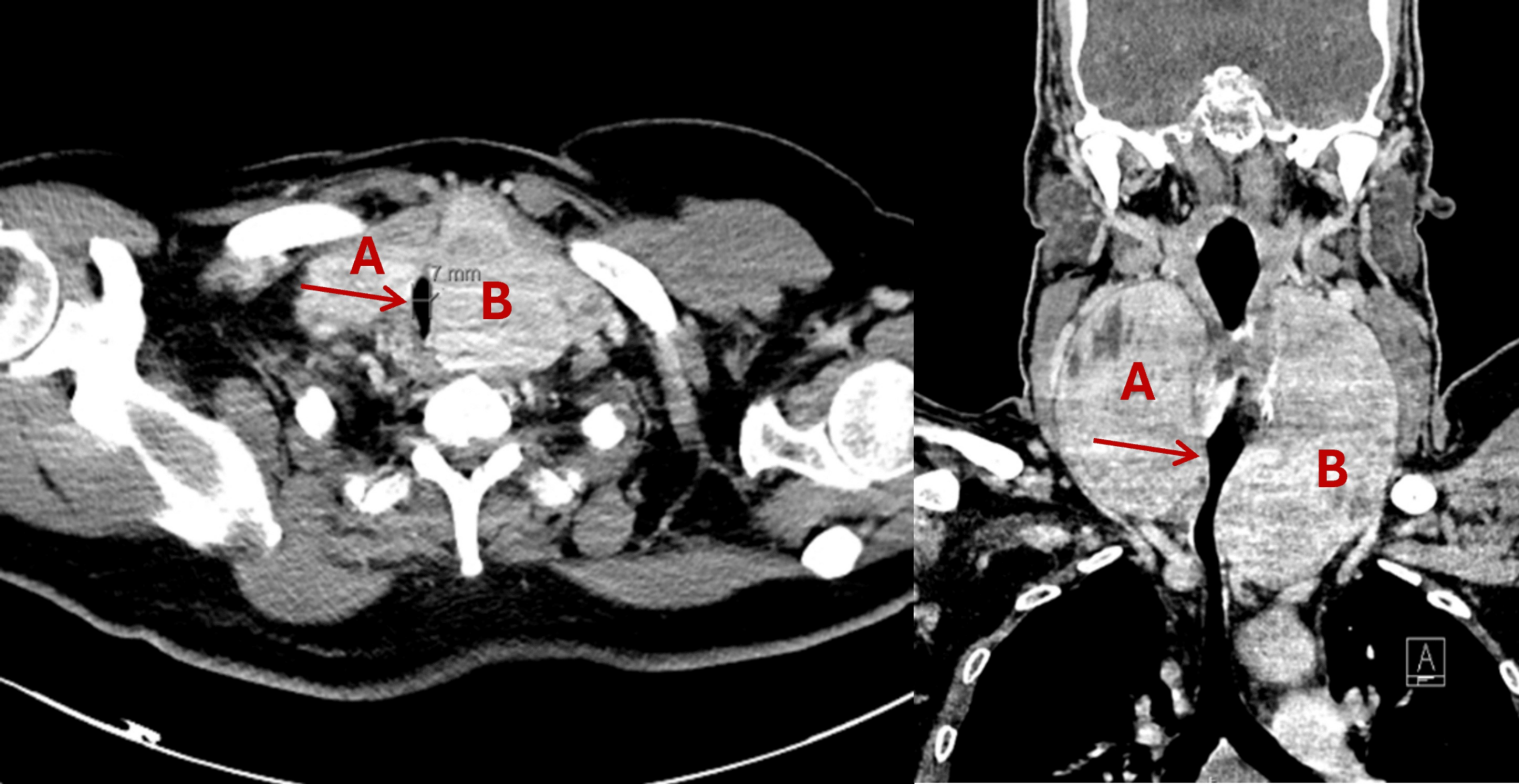
A CT scan of the patient’s neck in axial (left) and coronal (right) views A) Right thyroid lobe. (B) Left thyroid lobe. Arrows indicate tracheal lumen narrowing.

Contrast-enhanced CT of the neck and chest revealed a massive multinodular goiter with retrosternal extension, marked tracheal deviation, and severe tracheal narrowing with a minimum internal diameter of 6.2 mm over a compressed segment measuring approximately 3.5 cm in length. Axial and coronal CT images highlighting the extent of tracheal compression and asymmetrical thyroid enlargement are shown in Figure 1. Preoperative flexible nasopharyngolaryngoscopy demonstrated significant supraglottic narrowing with arytenoid redundancy, left hemilaryngeal paresis, and a poorly visualized glottic cleft. These findings indicated dynamic upper airway compromise and limited glottic patency, complementing the CT findings of severe infraglottic tracheal compression and further supporting the decision to pursue awake airway management. Preoperative laboratory testing confirmed euthyroid status and showed no clinically significant hematologic or renal abnormalities (Table 1).

**Table 1:** Preoperative laboratory evaluation demonstrating euthyroid status and normal baseline hematologic and renal function

Test	Result	Reference range
Thyroid-stimulating hormone (TSH)	2.07 µIU/mL	0.35-4.94 µIU/mL
Free thyroxine (free T4)	0.82 ng/dL	0.70-1.48 ng/dL
Hemoglobin	15.1 g/dL	13.0-18.0 g/dL
Platelet count	226 × 10⁹/L	150-400 × 10⁹/L
Serum creatinine	0.91 mg/dL	0.67-1.17 mg/dL

Given the anticipated difficult airway and the high likelihood of failure of emergency FONA due to distorted anatomy, a multidisciplinary plan was developed involving anesthesiology, otolaryngology, endocrine surgery, and perfusion services. AFOI was planned as the primary airway strategy, with rigid bronchoscopy and venovenous ECMO prepared as rescue options.

First awake intubation attempt

The first AFOI attempt was performed using topical airway anesthesia with lidocaine spray to the oropharynx and nasal passages. Sedation was intentionally avoided due to concern for airway obstruction and preservation of spontaneous ventilation. Supplemental oxygen was delivered via nasal cannula at 4 L/min. AFOI was performed with the patient in the sitting position to optimize airway patency and spontaneous ventilation. Despite patient cooperation, the procedure was poorly tolerated due to coughing, discomfort, and dynamic airway narrowing, preventing advancement of the bronchoscope beyond the compressed segment. The attempt was abandoned without hypoxemia or hemodynamic instability.

Reassessment and second awake intubation

Following reassessment, the decision was made to repeat AFOI with optimization of topicalization and technique rather than proceed with induction or alternative invasive strategies. Enhanced airway anesthesia was achieved using a combination of nebulized lidocaine, nasal mucosal preparation with vasoconstrictor and lidocaine gel, and lidocaine delivered to the supraglottic and glottic structures using a spray-as-you-go technique via a MADgic® atomization device. The total lidocaine dose remained within recommended safety limits (<9 mg/kg).

High-flow nasal oxygen was applied throughout the procedure. A flexible bronchoscope (3.9 mm) was advanced nasally, and a smaller diameter reinforced endotracheal tube (6.0 mm) was railroaded over the scope past the stenotic segment into the trachea. The patient remained spontaneously breathing throughout. Correct endotracheal tube placement was confirmed by bronchoscopy and continuous waveform capnography demonstrating stable end-tidal carbon dioxide values between 36 and 38 mmHg over multiple consecutive respiratory cycles, consistent with effective tracheal ventilation.

**Figure 2:**
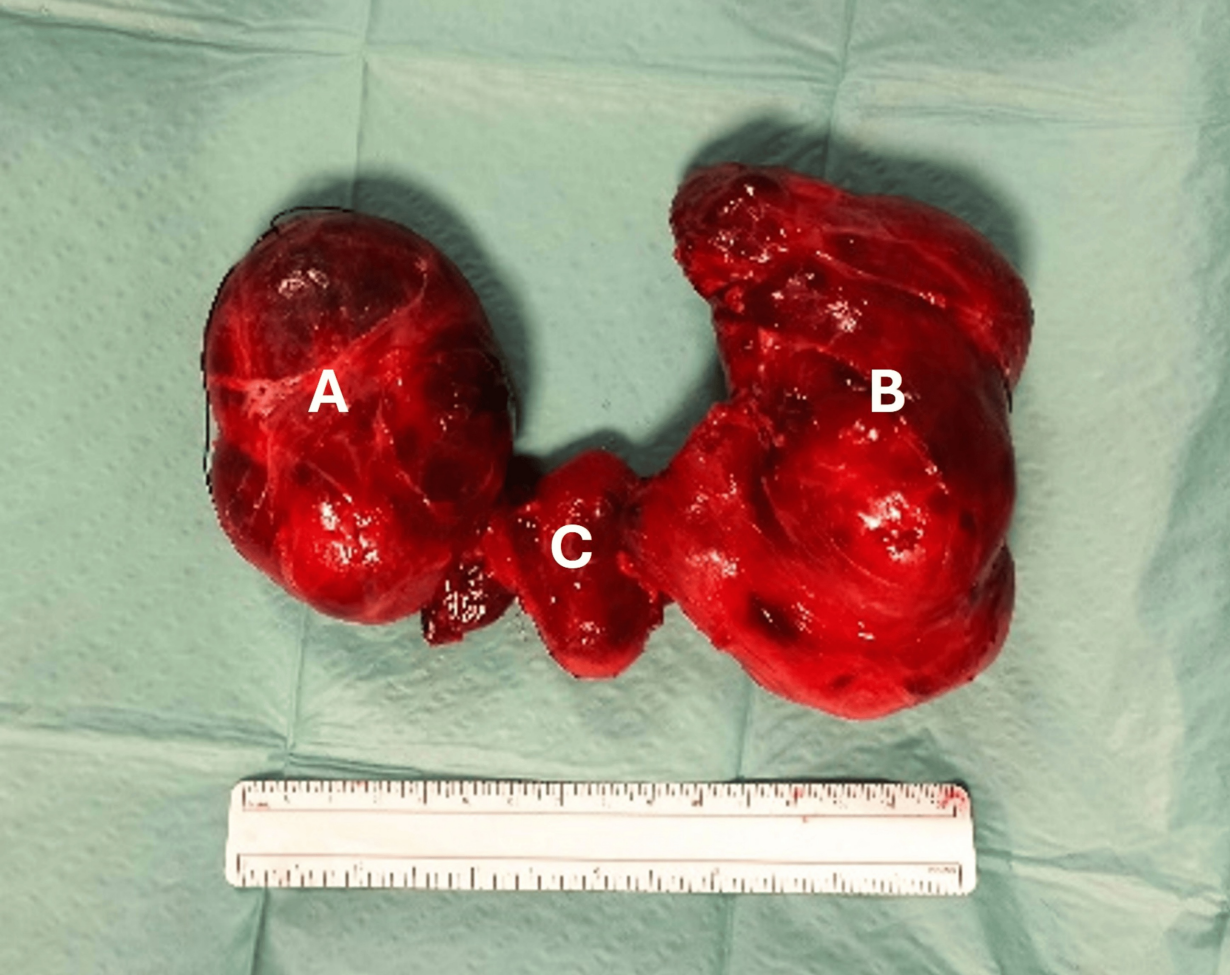
Thyroid gland after surgical removal A 15 cm ruler is shown for scale. (A) Right thyroid lobe. (B) Left thyroid lobe. (C) Isthmus.

General anesthesia was induced with intravenous fentanyl and propofol, followed by neuromuscular blockade with rocuronium. Maintenance of anesthesia was achieved with a volatile anesthetic (sevoflurane) administered in an oxygen-air mixture. Hemodynamic and respiratory parameters remained stable throughout induction and maintenance of anesthesia. Surgery proceeded uneventfully. Total thyroidectomy was concluded without tracheal injury. The excised specimen confirmed a large multinodular goiter (Figure 2).

Postoperative airway management and extubation

Given the severity and chronicity of tracheal compression, the patient was transferred intubated to the intensive care unit for close monitoring. Sedation with dexmedetomidine was used to facilitate comfort while maintaining spontaneous breathing.

**Figure 3:**
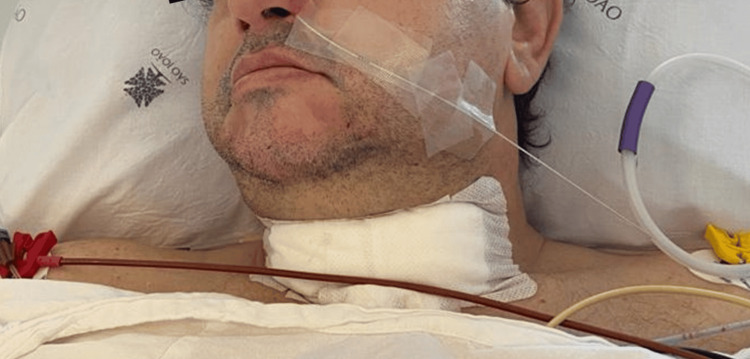
Cook® Airway Exchange Catheter on postoperative day 2

On postoperative day 2, flexible bronchoscopy showed improved tracheal lumen caliber with no evidence of dynamic airway collapse. A planned, staged extubation was therefore performed over a Cook® Staged Extubation Set (14 Fr, 83 cm) (Figure 3), providing continuous airway access in the event of respiratory compromise or need for reintubation. Supplemental oxygen via the catheter was not required. The patient remained stable, and the catheter was removed later that day. The patient was discharged home on postoperative day five without complications.

## Discussion

Large multinodular or substernal goiters can cause profound tracheal deviation and compression, creating high-risk airway scenarios that require meticulous preoperative planning. As tracheal luminal diameter decreases, the risk of airway obstruction and collapse increases, particularly following induction of general anesthesia [2,3]. Although thyroidectomy is the definitive treatment for compressive goiters, airway management often represents the most critical and hazardous phase of care, particularly when FONA is not feasible.

Radiologic parameters such as minimum tracheal diameter less than 8 mm and long-segment compression have been associated with increased risk of post-induction airway collapse, reinforcing the preference for awake airway techniques in patients with severe anatomical distortion [2,3]. AFOI allows preservation of spontaneous ventilation and airway tone while maintaining patient cooperation and physiologic stability.

Chronic tracheal compression can lead to pressure-induced weakening of cartilage and postoperative tracheomalacia. Although relatively rare, tracheomalacia may lead to airway collapse after extubation, necessitating delayed extubation or prolonged airway support [4,5].

Importantly, extubation in this context should be regarded as a second high-risk airway intervention. Post-thyroidectomy airway compromise may result from residual tracheomalacia, airway edema, hematoma, recurrent laryngeal nerve injury, or sudden loss of external tracheal support following gland removal. Recent airway management literature supports delayed extubation, bronchoscopic reassessment, and the use of airway exchange catheters to maintain continuous airway access and reduce extubation-related morbidity in high-risk patients [4-6].

In extreme cases where conventional airway strategies, including AFOI, videolaryngoscopy, and FONA, are predicted to fail, ECMO has emerged as a potential rescue modality. Several case reports describe ECMO-assisted airway management in patients with massive goiters causing critical tracheal obstruction [7,8]. Pre-emptive cannulation under local anesthesia can provide controlled oxygenation and ventilation during airway instrumentation or surgery [9]. However, ECMO carries significant risks, including bleeding, systemic inflammatory response, airway edema, and renal dysfunction, and should be reserved for selected cases in specialized centers. In the present case, pre-emptive ECMO was avoided due to its invasiveness and the absence of absolute indications at baseline.

## Conclusions

Severe tracheal compression from substernal multinodular goiter poses significant challenges for airway management. AFOI remains the safest and most reliable technique; however, failure may occur and should prompt reassessment rather than abandonment of the awake approach. Detailed preoperative evaluation, objective confirmation of airway placement, and careful planning for extubation are essential. In exceptional circumstances where conventional airway strategies are predicted to fail and FONA is not feasible, ECMO may serve as a life-saving rescue modality. Early multidisciplinary planning and vigilance throughout both induction and extubation are critical to optimizing patient outcomes.
